# Higher-dose maternal vitamin B₁₂ supplementation and infant neurodevelopmental subdomains: a secondary analysis of the MATCOBIND randomised controlled trial from India and Nepal

**DOI:** 10.1136/bmjph-2026-006254

**Published:** 2026-11-09

**Authors:** Jitender Nagpal, Swapnil Rawat, Ramasheesh Yadav, Manu Mathur, Rajendra Pant, Anita Shah, Atul Singhal, Mario Cortin Borja, Michelle Heys, Monica Lakhanpaul

**Affiliations:** 1 Sitaram Bhartia Institute of Science and Research, New Delhi, India; 2 Department of Clinical Epidemiology, Sitaram Bhartia Institute of Science and Research, New Delhi, India; 3 Queen Mary University of London Faculty of Medicine and Dentistry, London, UK; 4 Public Health Foundation of India, New Delhi, India; 5 Independent Researcher, Kathmandu, Kathmandu, Nepal; 6 Women Empowerment Society, Kathmandu, Nepal; 7 PPP, UCL, London, UK; 8 Population, Policy and Practice Programme, UCL Great Ormond Street Institute of Child Health, London, UK; 9 University College London; 10 UCL Great Ormond St. Institute of Child Health, London, UK; 11 General and Adolescent Paediatrics Unit, University College London, London, UK

**Keywords:** Community Health, Public Health, Primary Prevention

## Abstract

**Introduction:**

The MATCOBIND trial found a higher mental developmental quotient at 9–12 months in infants of mothers given 250 µg/day rather than 50 µg/day of vitamin B_₁₂_. We examined whether this difference was distributed across all mental developmental domains or concentrated in particular ones.

**Methods:**

Secondary analysis of a multicentre, double-blind randomised controlled trial conducted at two tertiary maternity centres in New Delhi (India) and Kathmandu (Nepal), including 531 mother–infant dyads (250 µg/day n=255; 50 µg/day n=276). Infants were assessed at 9–12 months with the Developmental Assessment Scale for Indian Infants. We compared the two arms on each subdomain that could be meaningfully scored at this age and on two composite groupings: a language grouping (domains VI and VII), the primary outcome and a cognition grouping (domains IV, V and VIII). Each domain score was expressed as a developmental age and as a quotient relative to the child’s age. Multiplicity was controlled using the Benjamini-Hochberg false discovery rate.

**Results:**

531 mother–infant dyads were assessed (250 µg/day n=255; 50 µg/day n=276). The language grouping favoured the higher dose: mean difference 2.80 quotient points (95% CI 0.95 to 4.65), and this was the only outcome to meet false discovery rate control on both co-primary representations (q=0.006 and q=0.026). Differences in the individual language domains did not survive adjustment for multiple comparisons (q=0.060). All motor domains, the cognition grouping and the remaining mental domains showed no clear evidence of between-arm differences. Estimates were consistent across sites (interaction p=0.484) and after adjustment for loss to follow-up.

**Conclusions:**

In this exploratory secondary analysis, the difference between arms was concentrated in language-related development. The effect is small, its clinical importance is not established, and the measures are derived rather than validated subscales.

**Trial registration number:**

NCT04083560.

**WHAT IS ALREADY KNOWN ON THIS TOPIC**

- Maternal vitamin B_₁₂_ deficiency is prevalent in South Asian pregnancies and has been linked in observational studies to poorer offspring cognitive and language development, but randomised trials of maternal supplementation have produced limited and inconsistent evidence that it improves these outcomes.
- The MATCOBIND trial showed that 250 µg/day methylcobalamin during pregnancy and early lactation improved infant mental developmental quotient at 9–12 months compared with 50 µg/day, without affecting motor developmental quotient; which neurodevelopmental subdomains accounted for this benefit was unknown.

**WHAT THIS STUDY ADDS**

- In an exploratory secondary analysis of MATCOBIND, not specified in the original trial protocol, the mental developmental quotient advantage appeared concentrated in language-related development rather than distributed across mental functions.
- The language composite was the only outcome to meet false discovery rate control; the individual language domains did not, and all motor domains and the cognition composite showed no clear evidence of a difference. The pattern is consistent with a language-centred effect.

**HOW THIS STUDY MIGHT AFFECT RESEARCH, PRACTICE OR POLICY**

- Future supplementation trials in populations with prevalent B_₁₂_ deficiency may wish to prespecify language outcomes and include validated measures of receptive and expressive language.
- If replicated, these findings would suggest that developmental surveillance relying on composite scores alone may be insensitive to domain-specific nutritional effects.
- Longer-term follow-up is needed to determine whether the early language-centred signal persists, and participant-level mediation analyses should clarify the role of maternal biochemical response.

## Introduction

 Vitamin B_₁₂_ supports folate-mediated one-carbon metabolism, DNA synthesis and methylation and is relevant to fetal growth and neurodevelopment.^1,2^ In South Asia and other populations with low intake of animal-source foods, biochemical B_₁₂_ deficiency in pregnancy is common. Indian data suggest 40–70% of pregnant women may be deficient,^3,4^ a burden confirmed by recent data.^5^ Recent reviews conclude that the observational associations between maternal vitamin B_₁₂_ status and offspring neurodevelopment are suggestive but heterogeneous, and that randomised evidence on whether maternal supplementation improves offspring cognitive or language outcomes remains limited in quantity and inconsistent in direction.^6–8^

Previous maternal supplementation trials in South India did not uniformly improve early infant neurodevelopment, although later follow-up suggested a benefit in expressive language.^9,10^ The PRIYA preconception trial reported improved offspring cognition and language at 2 years among children whose mothers received B_₁₂_ alone.^11^ Observational cohorts have been broadly consistent. In the Singaporean GUSTO pregnancy cohort, lower maternal plasma B_₁₂_ during pregnancy was associated with poorer infant cognitive outcomes at 24 months.^12^ The Spanish ECLIPSES study reported associations between maternal B_₁₂_ status during pregnancy and early infant neurodevelopment.^13^ Together these data suggest that, in populations with prevalent deficiency, maternal B_₁₂_ supplementation can reliably improve biochemical status, while developmental benefits may be dose, duration and/or domain specific. Recent reviews converge on language as the developmental domain most consistently sensitive to maternal B_₁₂_ status, even where global cognitive effects appear modest.^6–8^

The MATCOBIND trial addressed this question in a nutritionally vulnerable, predominantly vegetarian population in India and Nepal by comparing 250 µg/day with 50 µg/day methylcobalamin from early pregnancy until 6 months post partum.^14,15^ Infants in the higher-dose group had a higher mean mental developmental quotient (DQ) at 9–12 months, whereas motor DQ did not differ.^15^ The trial also showed larger increases in maternal serum vitamin B_₁₂_ and holotranscobalamin in the higher-dose group, while infant anthropometry and neonatal morbidity were broadly similar between groups.^15^

To further understand if the benefit observed was specific to subdomains like language or a more diffuse change, we undertook a secondary exploratory analysis of data from the MATCOBIND trial. We also aimed to determine if the changes were directionally concordant between study sites.

## Methods

### Design, setting and participants

This study analyses data from the MATCOBIND trial (Clinical Trials Registry of India, CTRI/2018/07/015048; ClinicalTrials.gov, NCT04083560), a multicentre, double-blind, parallel-group randomised controlled trial of oral methylcobalamin supplementation (250 µg/day vs 50 µg/day) in predominantly vegetarian pregnant women, conducted at two tertiary maternity care centres in New Delhi, India (pay-for-care facility catering to middle income groups) and Kathmandu, Nepal (free care catering predominantly to lower income groups). The study protocol, trial eligibility, randomisation, masking, intervention, the biochemical assessment schedule and the primary analysis are described elsewhere.^14,15^ The analytical sample for this secondary analysis comprised the 531 mother–infant dyads with DASII follow-up at 9–12 months (250 µg/day n=255; 50 µg/day n=276).^15^ The parent-trial mental DQ and motor DQ findings at 9–12 months were treated as the primary outcomes of MATCOBIND.^15^

### Neurodevelopmental assessment

Infant neurodevelopment at 9–12 months in the MATCOBIND trial was assessed using the Developmental Assessment Scale for Indian Infants (DASII), a locally standardised infant developmental scale widely used in Indian clinical and research settings.^16^ Assessments were performed by trained developmental assessors blinded to allocation.^15^ Corrected age was used for preterm infants and chronological age for term infants. Total raw scores were converted to mental DQ and motor DQ using standard DASII procedures.^15^

### DASII administration across sites

The DASII, an adaptation of the Bayley Scales of Infant Development standardised on Indian infants,^16^ was used at both sites so that infants in both populations were assessed with a single instrument and common norms, as in the parent trial.^15^ The Bayley-III has been culturally adapted and evaluated in Nepal,^17^ but published experience with the DASII there is limited to its use at the Kathmandu site in this trial and in one subsequent cohort.^18^ The DASII is administered by a trained examiner and scored on the infant’s observed performance; the language items assess vocalisation, response to and comprehension of speech and word production, elicited in the language spoken at home, and no translated test form was required. Developmental therapists from both sites were trained together at trial onset, understood Hindi and English, the languages of the manual and were blinded to allocation. Inter-rater agreement in joint assessments gave an intraclass correlation coefficient of 0.89; a video-reviewed pilot (n=10) gave 0.93 and a mid-trial repeat (n=10) gave 0.96.^15^

DASII items are grouped into 5 motor and 10 mental content domains.^16^ These are item-level content clusters describing developmental profile rather than independently normed psychometric subscales, and domain totals do not support standardised interpretation in their raw form.^16^ To overcome this, we identified four complementary representations of domain performance:

- RAW domain scores (count of items passed).
- Age-expected attainment ratio (AEAR): (items passed for corrected age/items expected for corrected age)×100.
- Performance-age: performance age outputs for domain specific items passed from DASII workbook mapping tables.
- Subdomain quotient: (performance-age/age at assessment)×100.

Corrected age was used for infants born preterm and chronological age for infants born at term, in the DASII scoring procedure, in the denominator of the AEAR and in the denominator of the Subdomain quotient.

Domains were excluded from inferential analysis in advance where more than 95% of infants scored at the maximum or minimum attainable value, where fewer than five items were scorable at this age, or where scores showed negligible between-infant variability. Mental I (visual cognisance) was excluded on the last criterion: although 90.2% of infants were at the maximum, below the 95% threshold, only three distinct scores were observed. Ceiling and floor proportions for all 15 domains are given in online supplemental table S1. The analysable motor subdomains were therefore Body control (II), Locomotion-I (III) and Manipulation (V), and the analysable mental subdomains were Reaching and manipulation (III), Memory (IV), Social interaction and imitative behaviour (V), Language 1 (vocalisation, speech and communication; VI), Language 2 (vocabulary and comprehension; VII) and Understanding relationships (VIII).

Further, noting the limited number of items (and score diversity) in many of the individual domains and to reduce multiplicity, two composites were formed to summarise correlated developmental families. The language composite (VI-VII) was the primary outcome. The cognition composite (IV-V-VIII: memory, social interaction and imitation and understanding relationships) was a key supportive composite. For the raw score, performance-age and Subdomain quotient representations, each composite is the unweighted arithmetic mean of the component domain values for that infant; the language composite Subdomain quotient, for example, is the mean of the Subdomain quotients for domains VI and VII. For the AEAR representation the composite is an item-weighted pooled ratio: the total number of items passed across the constituent domains divided by the total number of items expected for the infant’s age across those domains, multiplied by 100.

### Analytical framework

The full analytical framework, including which domains feed into the composites, is shown in figure 1. Before any group-level analysis, the investigator team agreed that the DASII content domains and their developmental interpretation would form the analytical framework; that domains would be admitted to inferential analysis only on the basis of allocation-masked pooled distributions, excluding any with a ceiling or floor effect or fewer than five scorable items in the 9–12 months window, applied identically to motor and mental domains; that domains would be aggregated into developmentally coherent composites, with multiplicity controlled within a motor family, a mental family and a composite family; that language would be the primary theme of interest; and that the false discovery rate (FDR) would be controlled by the Benjamini-Hochberg (BH) procedure at q<0.05. The domain exclusions, the families and the composites were then fixed at a second meeting from the masked distributions, with the analyst blinded to allocation until that point. The representations, including the Subdomain quotient and its co-primary pairing with performance-age, and the final membership of the cognition composite were settled during the analysis; the language composite (domains VI and VII) was unchanged throughout.

**Figure 1:**
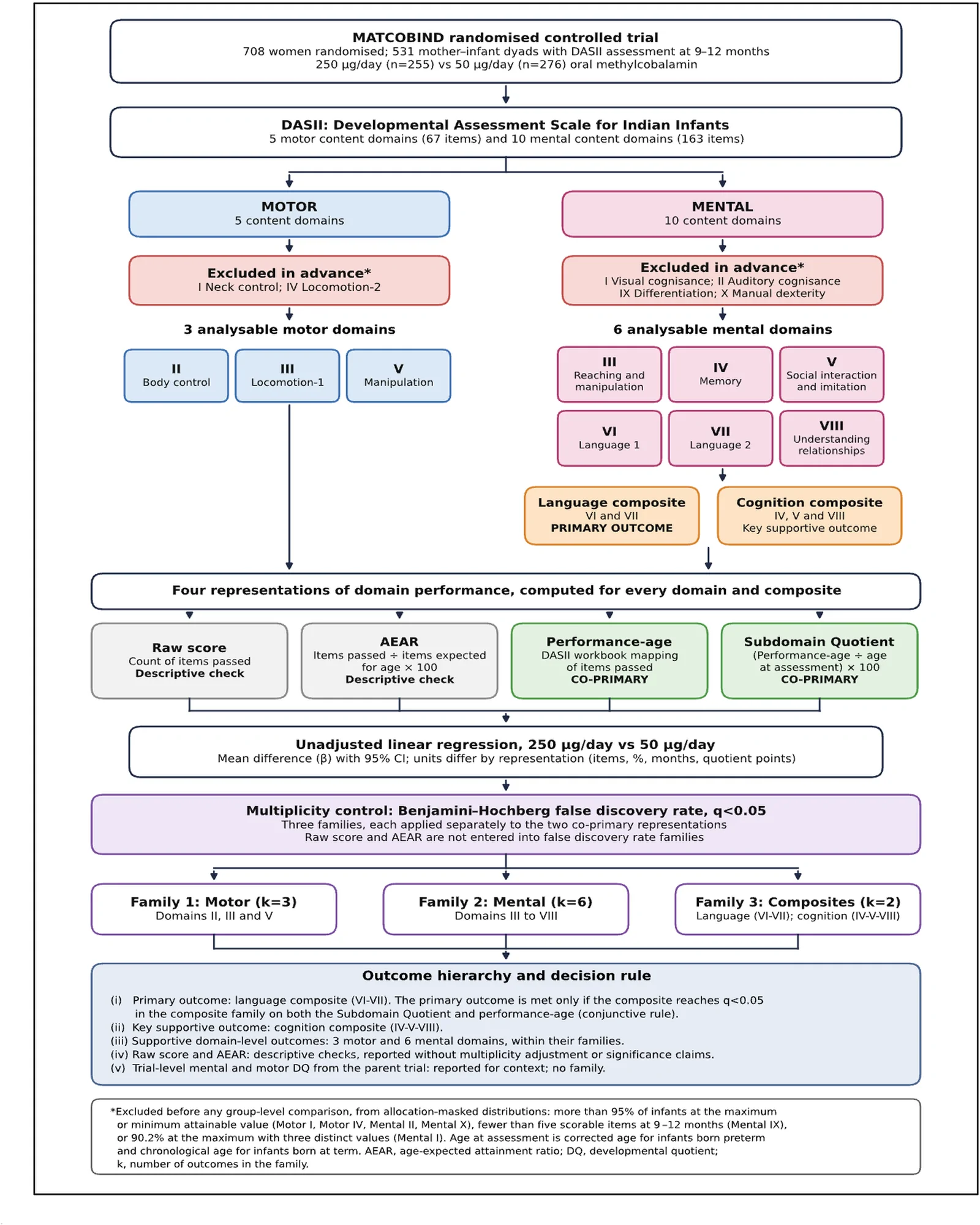
Analytical framework for the MATCOBIND DASII subdomain analysis (n=531). The schematic shows the outcome hierarchy (primary outcome, co-primary representations, descriptive checks), the DASII motor and mental domains, domains excluded a priori at 9–12 months because of ceiling/floor effects, negligible between-infant variability (Mental I) or insufficient scorable items, the analysable motor (n=3) and mental (n=6; III-VIII) domains and the two composites (VI-VII language; IV-V-VIII cognition). Four representations are shown: raw score (items passed), AEAR, performance-age and Subdomain quotient. Benjamini-Hochberg false discovery rate families used for multiplicity control (motor, mental III-VIII and composites) are indicated. DASII, Developmental Assessment Scale for Indian Infants.

The primary outcome was the language composite (mental domains VI and VII). Its co-primary representations were the Subdomain quotient and performance-age. The primary contrast was the unadjusted mean difference (250 µg/day minus 50 µg/day). The cognition composite (mental domains IV, V and VIII) was the key supportive outcome, and the three analysable motor domains and six analysable mental domains (III to VIII) were supportive domain-level outcomes, with BH control within the motor family (k=3) and the mental family (k=6) on each co-primary representation. Raw score and AEAR were descriptive checks, reported without multiplicity adjustment and without significance claims. Maternal biochemical changes during pregnancy and infant biomarker outcomes from the parent trial^15^ were used to provide biological context.

### Patient and public involvement

Patient and public involvement informed the design and conduct of the parent MATCOBIND trial, from which this secondary analysis draws. A panel of eight parents (four couples, two from each site, all of whom had delivered within the preceding 2 years) reviewed and helped refine participant-facing materials, the consent and recruitment processes and the follow-up schedule; one parent pair continued on the trial oversight committee to provide ongoing lay perspectives. Exploratory qualitative interviews with pregnant women at both sites identified facilitators of and barriers to participation, including low awareness of vitamin B_₁₂_ but willingness to engage, alongside concerns about supplement side effects, mid-trial relocation and out-of-pocket costs. These insights led to concrete protocol changes: B_₁₂_ educational materials, a revised consent pathway in which the routine antenatal clinician introduced the study before detailed consent, weekly reminder messages, reimbursement of travel and test expenses and culturally adapted study instruments. Participants were not involved in the design of this secondary analysis or in the present interpretation of subdomain-level findings. Results will be disseminated to participants and the public through lay summaries and community engagement activities.

### Statistical analysis

Differences between groups were estimated using unadjusted linear regression and reported as mean differences (β) with 95% CIs and p values. Units of β depended on the representation: items passed for raw scores, percentage points for AEAR, months for performance-age and quotient points for the Subdomain quotient. Multiplicity was controlled using the B procedure^19^ at an FDR of q<0.05 within three families, the composite family (language and cognition, k=2), the mental domain family (domains III to VIII, k=6) and the motor domain family (domains II, III and V, k=3), applied separately to each of the two co-primary representations. The decision rule for the primary outcome was conjunctive: the analysis was regarded as supporting a language-centred effect only if the language composite reached q<0.05 in the composite family on both the Subdomain quotient and performance-age. Because both tests must succeed, this rule controls the type I error at the nominal level without further adjustment.^20^ Trial-level mental and motor DQ are reported for context and belong to no family. Raw score and AEAR are deterministic re-expressions of the same item responses and are reported without multiplicity adjustment as descriptive checks.

Sample size was predetermined by the number of mother–infant dyads with DASII follow-up in the parent trial (n=531).

Missing outcome data. The analytical cohort comprised the mother–infant dyads who completed DASII assessment at 9–12 months. All infants in the analytical cohort had complete values for every analysed domain, composite and representation, so all analyses are complete-case analyses with no within-cohort imputation. Baseline characteristics of women included and not included were compared and, to assess the potential effect of loss to follow-up, the primary contrast was re-estimated, on the Subdomain quotient only, with inverse probability of inclusion weights and again after multiple imputation.^21^

Sensitivity analyses: Site-stratified estimates were obtained for every row of figure 2, and for each the treatment-by-site interaction was tested by adding a treatment×site term to the regression model; these tests are descriptive and unadjusted for multiplicity, and only that for the language composite is interpreted.^22^

**Figure 2:**
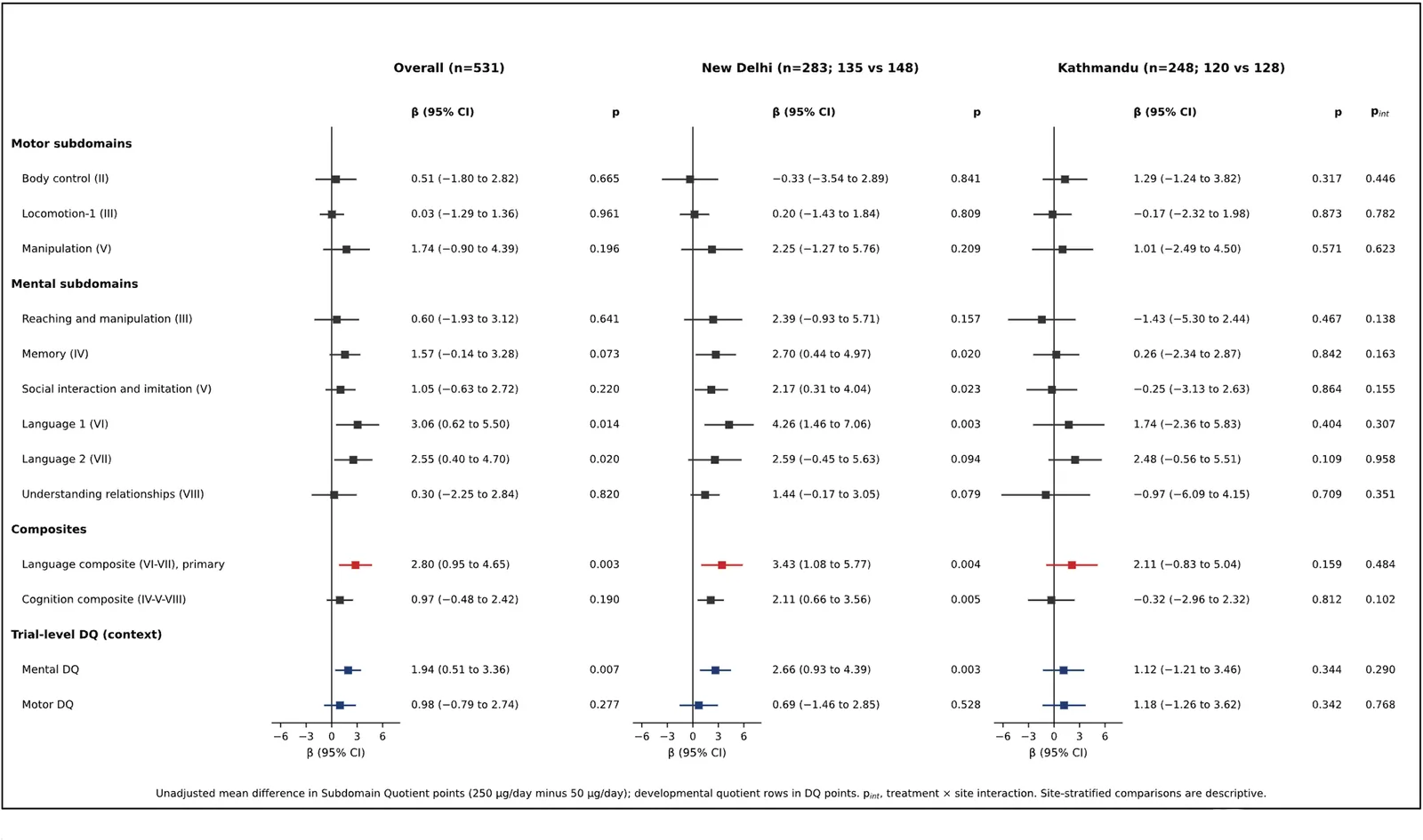
Forest plot of unadjusted mean differences (β) comparing 250 µg/day with 50 µg/day on the Subdomain quotient representation. Estimates are shown for the overall cohort and stratified by site (India and Nepal). Points indicate β and horizontal bars 95% CIs; the vertical line indicates no difference (β=0). Rows include the analysable motor and mental subdomains, the two composites (VI-VII language; IV-V-VIII cognition) and trial-level mental and motor DQ for context. p_int_ denotes the site-by-treatment interaction p value from a model including a treatment×site term; site-stratified comparisons are descriptive. DQ, developmental quotient.

Analyses were performed in Stata V.17 (StataCorp LLC, College Station, Texas, USA). Mean differences were estimated by linear regression (regress); BH q values were computed from the ranked unadjusted p values within each family; inverse probability weighting used a logistic model for inclusion with robust SEs, and multiple imputation used chained equations (mi impute chained; m=200).

## Results

### Participants and baseline characteristics

The parent MATCOBIND trial randomised 708 women; 531 mother–infant dyads (75.0%) had DASII neurodevelopmental follow-up at 9–12 months (250 µg/day n=255; 50 µg/day n=276).^15^ The trial showed that infants in the 250 µg/day group had a higher mean mental DQ than infants in the 50 µg/day group (103.7 (SD 7.7) vs 101.7 (SD 8.8); p=0.007), whereas motor DQ did not differ (98.3 (9.9) vs 97.3 (10.7); p=0.277).^15^ Baseline characteristics of the mothers and infants contributing to this analysis are shown in table 1. No hypothesis tests of baseline balance are reported.^23^ Post-randomisation infant feeding characteristics are reported here rather than in table 1: exclusive breastfeeding at 3 months, recorded for the 264 dyads whose mothers attended the 3-month visit, was 99/124 (79.8%) in the 250 µg/day group and 120/140 (85.7%) in the 50 µg/day group, and breastfeeding at 6 months, recorded for 237 dyads, was 91/111 (82.0%) and 110/126 (87.3%).

**Table 1:** Baseline characteristics of the mothers and infants in the analytical cohort (n=531; 250 µg/day n*=*255, 50 µg/day n*=*276)

Characteristic	250 µg/day (n=255)	50 µg/day (n=276)
Maternal characteristics at enrolment		
New Delhi site, n (%)	135 (52.9)	148 (53.6)
Age, years, mean (SD)	27.89 (4.68)	28.31 (4.53)
Head-of-household education, graduate or above, n (%)*	141 (55.3)	158 (57.2)
Primigravida, n (%)	159 (62.4)	159 (57.6)
Gestational age at enrolment, weeks, mean (SD)	9.16 (1.97)	9.01 (1.93)
Pre-pregnancy body mass index, kg/m², mean (SD)	23.17 (3.93)	23.40 (3.64)
Height, cm, mean (SD)	156.89 (5.69)	156.70 (6.40)
No meat or fish intake, n (%)	199 (78.0)	195 (70.7)
Serum vitamin B_₁₂_, pg/mL, mean (SD)	217.50 (112.75)	230.00 (121.43)
Homocysteine, µmol/L, mean (SD)	11.59 (6.85)	11.15 (6.59)
Holotranscobalamin, pmol/L, mean (SD)	37.06 (29.97)	41.13 (33.89)
Haemoglobin, g/L, mean (SD)	122.71 (11.35)	121.43 (12.30)
Newborn and infant characteristics		
Male, n (%)	135 (52.9)	124 (45.1)
Mode of delivery, n (%)		
Normal vaginal	161 (63.4)	191 (69.5)
Operative vaginal	17 (6.7)	11 (4.0)
Caesarean	76 (29.9)	73 (26.5)
Birth weight, g, mean (SD)	3043.1 (441.6)	3010.8 (414.4)
Birth length, cm, mean (SD)	48.9 (2.4)	48.8 (2.2)
Head circumference, cm, mean (SD)	34.2 (1.5)	34.0 (1.3)
APGAR at 1 min, mean (SD)	7.4 (1.4)	7.4 (1.4)
APGAR at 5 min, mean (SD)	8.4 (0.8)	8.4 (0.7)
APGAR at 10 min, mean (SD)	9.1 (0.5)	9.1 (0.4)
Corrected age at assessment, months, mean (SD)	9.7 (0.8)	9.7 (0.8)

Values are n (%) or mean (SD) for the 531 mother–infant dyads in the analytical cohort (250* *µg/day n=255; 50* *µg/day n=276). Sex was not recorded for one infant in the 50 µg/day group. Maternal characteristics were recorded at enrolment in the first trimester; laboratory values are first-trimester serum concentrations. Consistent with CONSORT guidance, no hypothesis tests of baseline balance are reported. Post-randomisation infant feeding characteristics are reported in the Results, and women included and not included in this analysis are compared in online supplemental table S2.

* Head-of-household education classified per the Kuppuswamy scale of socioeconomic status.

CONSORT, Consolidated Standards of Reporting Trials.

Women whose infants did not undergo DASII assessment had poorer baseline vitamin B_₁₂_ status than those who did, whereas socio-demographic and anthropometric characteristics were similar (online supplemental table S2).

In the trial, maternal serum vitamin B_₁₂_ rose by a median of 105.0 pg/mL in the 250 µg/day group versus 54.0 pg/mL in the 50 µg/day group during pregnancy (p<0.001), and holotranscobalamin rose by 47.4 versus 32.9 pmol/L (p<0.001). Third-trimester maternal homocysteine was lower in the 250 µg/day group, whereas changes in ferritin, folate and vitamin D did not differ between groups.^15^ At infant follow-up (9–12 months), infant serum B_₁₂_ and holotranscobalamin did not differ between randomised groups.^15^

### Motor subdomains

All three analysable motor domains-body control, locomotion and manipulation had point estimates close to the line of no effect, with 95% CIs spanning the null. The pattern was consistent across representations (table 2). Within the motor FDR family, no domain reached q<0.05 on any representation and the Manhattan plot (figure 3) shows the motor rows lying below both the conventional p=0.05 line and the BH-FDR threshold across all representations. The parent-trial null motor DQ result was therefore preserved at the domain level.

**Table 2:** Between-group differences in DASII subdomains and composites at 9–12 months (Group A, 250 µg/day vs Group B, 50 µg/day)

Domain/composite/DQ	Representation	Group A 250 µg/day, mean (SD)	Group B 50 µg/day, mean (SD)	β (95% CI)	P value	Q value
Trial-level developmental quotient						
Mental DQ	Developmental quotient	103.7 (7.7)	101.7 (8.8)	1.94 (0.51 to 3.36)	0.007	NA
Motor DQ	Developmental quotient	98.3 (9.9)	97.3 (10.7)	0.98 (−0.79 to 2.74)	0.277	NA
Motor subdomains						
Body control (II)	Raw score	19.1 (1.2)	19.1 (1.2)	0.03 (−0.18 to 0.24)	0.780	NA
	AEAR	100.5 (6.7)	100.2 (6.8)	0.37 (−0.78 to 1.52)	0.528	NA
	Performance-age	9.2 (1.3)	9.2 (1.3)	0.03 (−0.19 to 0.25)	0.787	0.787
	Subdomain quotient	95.2 (13.6)	94.7 (13.5)	0.51 (−1.80 to 2.82)	0.665	0.961
Locomotion-I (III)	Raw score	4.1 (0.3)	4.1 (0.5)	−0.02 (−0.09 to 0.06)	0.688	NA
	AEAR	81.3 (6.2)	81.5 (10.4)	−0.12 (−1.60 to 1.36)	0.869	NA
	Performance-age	7.6 (0.5)	7.6 (0.8)	−0.03 (−0.15 to 0.10)	0.682	0.787
	Subdomain quotient	78.6 (6.9)	78.5 (8.5)	0.03 (−1.29 to 1.36)	0.961	0.961
Manipulation (V)	Raw score	13.6 (0.8)	13.5 (0.9)	0.07 (−0.07 to 0.21)	0.320	NA
	AEAR	105.6 (7.4)	104.8 (7.8)	0.83 (−0.47 to 2.13)	0.210	NA
	Performance-age	10.7 (1.4)	10.6 (1.4)	0.15 (−0.10 to 0.39)	0.234	0.703
	Subdomain quotient	110.9 (15.1)	109.1 (15.9)	1.74 (−0.90 to 4.39)	0.196	0.589
Mental subdomains						
Reaching and manipulation (III)	Raw score	27.3 (1.1)	27.3 (1.2)	0.06 (−0.14 to 0.25)	0.571	NA
	AEAR	100.2 (3.7)	100.1 (4.2)	0.15 (−0.52 to 0.82)	0.651	NA
	Performance-age	7.3 (1.7)	7.2 (1.7)	0.05 (−0.24 to 0.34)	0.740	0.888
	Subdomain quotient	74.3 (14.7)	73.7 (14.8)	0.60 (−1.93 to 3.12)	0.641	0.770
Memory (IV)	Raw score	9.4 (0.7)	9.3 (0.7)	0.10 (−0.03 to 0.22)	0.126	NA
	AEAR	102.9 (8.9)	101.4 (9.4)	1.47 (−0.09 to 3.04)	0.065	NA
	Performance-age	9.4 (0.8)	9.3 (0.9)	0.13 (−0.02 to 0.27)	0.082	0.164
	Subdomain quotient	97.7 (9.9)	96.1 (10.2)	1.57 (−0.14 to 3.28)	0.073	0.145
Social interaction (V)	Raw score	15.0 (1.7)	14.8 (1.7)	0.17 (−0.13 to 0.46)	0.265	NA
	AEAR	116.1 (13.7)	115.7 (14.0)	0.46 (−1.90 to 2.82)	0.702	NA
	Performance-age	10.7 (0.9)	10.7 (1.0)	0.07 (−0.10 to 0.23)	0.420	0.630
	Subdomain quotient	110.9 (9.2)	109.9 (10.3)	1.05 (−0.63 to 2.72)	0.220	0.329
Language 1 (VI)	Raw score	6.7 (0.7)	6.5 (0.8)	0.15 (0.02 to 0.28)	0.022	NA
	AEAR	105.1 (12.2)	102.9 (12.9)	2.24 (0.10 to 4.38)	0.040	NA
	Performance-age	9.3 (1.4)	9.1 (1.5)	0.27 (0.02 to 0.51)	0.031	0.155
	Subdomain quotient	96.4 (13.8)	93.4 (14.7)	3.06 (0.62 to 5.50)	0.014	0.060
Language 2 (VII)	Raw score	3.0 (1.2)	2.8 (1.2)	0.16 (−0.05 to 0.37)	0.128	NA
	AEAR	103.6 (45.1)	97.2 (45.7)	6.32 (−1.43 to 14.07)	0.110	NA
	Performance-age	9.1 (1.2)	8.8 (1.4)	0.22 (−0.001 to 0.45)	0.052	0.155
	Subdomain quotient	93.3 (11.0)	90.8 (13.9)	2.55 (0.40 to 4.70)	0.020	0.060
Understanding relationships (VIII)	Raw score	1.0 (0.3)	1.0 (0.3)	0.00 (−0.05 to 0.05)	0.930	NA
	AEAR	98.0 (28.7)	97.8 (26.9)	0.21 (−4.52 to 4.95)	0.930	NA
	Performance-age	6.2 (1.5)	6.2 (1.4)	0.02 (−0.23 to 0.27)	0.895	0.895
	Subdomain quotient	64.5 (15.3)	64.2 (14.5)	0.30 (−2.25 to 2.84)	0.820	0.820
Composites						
Language (VI-VII; primary)	Raw score	4.8 (0.8)	4.7 (0.8)	0.15 (0.01 to 0.29)	0.030	NA
	AEAR	103.3 (17.8)	99.7 (18.8)	3.57 (0.45 to 6.70)	0.025	NA
	Performance-age	9.2 (1.1)	9.0 (1.2)	0.25 (0.05 to 0.44)	0.013	0.026
	Subdomain quotient	94.9 (10.0)	92.1 (11.5)	2.80 (0.95 to 4.65)	0.003	0.006
Cognition (IV-V-VIII; supportive)	Raw score	8.4 (0.7)	8.4 (0.7)	0.09 (−0.04 to 0.21)	0.162	NA
	AEAR	109.9 (9.4)	109.0 (9.8)	0.90 (−0.73 to 2.54)	0.279	NA
	Performance-age	8.8 (0.7)	8.7 (0.8)	0.07 (−0.06 to 0.20)	0.294	0.294
	Subdomain quotient	91.0 (8.2)	90.1 (8.8)	0.97 (−0.48 to 2.42)	0.190	0.190

Estimates are unadjusted mean differences (β) from linear regression with 95% CIs. Q values are Benjamini-Hochberg false discovery rate-adjusted p values within the motor (k=3), mental (k=6) and composite (k=2) families, computed separately for the two co-primary representations (Subdomain quotient and performance-age); raw score and AEAR are descriptive representations and were not entered into false discovery rate families (NA). Units: DQ points for mental DQ and motor DQ; items passed for raw scores; percentage points for AEAR; months for performance-age; quotient points for Subdomain quotient. Mental DQ and motor DQ are reproduced from the parent MATCOBIND trial and belong to no family. The six domains excluded in advance and the reasons for exclusion are given in online supplemental table S1.

AEAR, age-expected attainment ratio; DQ, developmental quotient.

**Figure 3:**
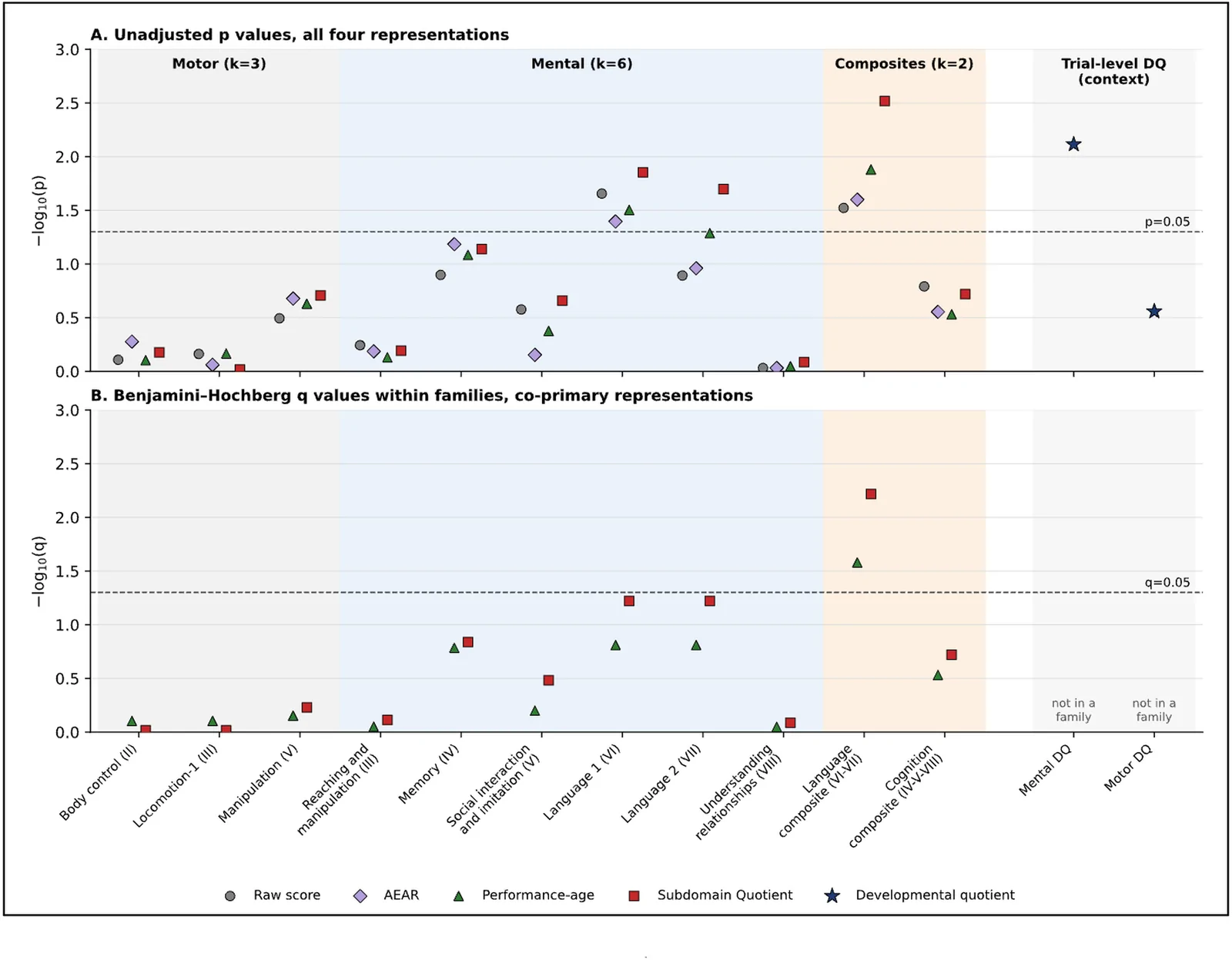
Manhattan-style plot summarising evidence across outcome representations. (A) Unadjusted −log₁₀(p) values; (B) −log₁₀(q) values after Benjamini-Hochberg false discovery rate adjustment within families (motor domains, mental III-VIII domains and composites), applied separately to each of the two co-primary representations (performance-age and Subdomain quotient); raw score and AEAR are not entered into false discovery rate families. The horizontal reference lines denote p=0.05 (A) and q=0.05 (B). Outcomes displayed include the three analysable motor domains, six analysable mental domains (III-VIII), the two composites (VI-VII language and IV-V-VIII cognition) and trial-level mental and motor DQ for context. AEAR, age-expected attainment ratio; DQ, developmental quotient.

### Mental subdomains

As presented in Figure 2, table 2, between-group differences for the mental domains favoured the 250 µg/day group, with the largest differences in the language domains. Language 1 (VI) was higher in the 250 µg/day group on both co-primary representations (Subdomain quotient β=3.06, 95% CI 0.62 to 5.50; p=0.014; performance-age β=0.27, 95% CI 0.02 to 0.51; p=0.031), with the descriptive raw score and AEAR estimates in the same direction (Table 2). Language 2 (VII) was higher on the Subdomain quotient (β=2.55, 95% CI 0.40 to 4.70; p=0.020) but not on performance-age (β=0.22, 95% CI −0.001 to 0.45; p=0.052); the raw score and AEAR estimates were of consistent sign. Memory (IV) and Social interaction and imitation (V) were directionally favourable to the intervention arm on each representation but did not attain individual statistical significance. Effects on Reaching and manipulation and Understanding relationships were null, with point estimates close to zero and confidence intervals spanning the line of no effect. No individual mental domain retained q<0.05 after FDR control (Table 2).

### Composites

As presented in table 2, figures 2 and 3, the VI-VII language composite, the primary outcome, was higher in the 250 µg/day group on both co-primary representations: Subdomain quotient β=2.80 (95% CI 0.95 to 4.65; p=0.003) and performance-age β=0.25 (95% CI 0.05 to 0.44; p=0.013); the descriptive raw score (β=0.15, 95% CI 0.01 to 0.29; p=0.030) and AEAR (β*=*3.57, 95% CI 0.45 to 6.70; p=0.025) estimates were in the same direction. The supportive IV-V-VIII cognition composite was directionally favourable across representations but did not reach statistical significance on any (raw β=0.09, 95% CI −0.04 to 0.21; performance-age β=0.07, 95% CI −0.06 to 0.20; Subdomain quotient β=0.97, 95% CI −0.48 to 2.42). The multiplicity-adjusted results are depicted in (B) of the Manhattan plot (figure 3): the language composite was the only outcome to cross the q=0.05 threshold, and it did so on both the Subdomain quotient (q=0.006) and performance-age (q=0.026) representations, satisfying the conjunctive decision rule. The standardised effect size for the language composite Subdomain quotient was 0.26 (95% CI 0.09 to 0.43).

### Sensitivity analyses

Site-stratified estimates are displayed alongside the overall estimates in figure 2, with the treatment-by-site interaction for each row. Estimates for the language domains and the language composite were in the same direction at both sites. For the language composite the interaction was 1.32 quotient points (95% CI −2.39 to 5.03; p=0.484), so these data provide no evidence that the effect differs between New Delhi and Kathmandu. Within-site p values are displayed for completeness but are not interpreted as evidence of heterogeneity, and the trial was not powered for interaction testing.^22^ The language composite estimate was similar with inverse probability of inclusion weighting (β=2.82, 95% CI 0.99 to 4.66; p=0.003) and after multiple imputation (β=2.80, 95% CI 0.93 to 4.66; p=0.003) (online supplemental table S3).

### Multiplicity adjustment

The multiplicity-adjusted picture across the analysis is summarised in (B) of the Manhattan plot (figure 3) and in online supplemental table S4. Within the domain families (three motor; six mental III-VIII), evaluated separately for each co-primary representation, no individual motor or mental domain reached q<0.05; Language 1 and Language 2 gave q=0.060 on the Subdomain quotient and q=0.155 on performance-age. Within the composite family, the VI-VII language composite met the threshold on both the Subdomain quotient (q=0.006) and performance-age (q=0.026). The IV-V-VIII cognition composite did not (q=0.19 and q=0.29).

## Discussion

The infant mental DQ advantage observed in the parent trial^15^ was accompanied by a pattern of subdomain differences concentrated in the language cluster (domains VI and VII), with estimates close to the null in the motor domains and in the remaining mental domains. This suggests a language-centred pattern rather than a diffuse benefit. The study was not designed to detect differential effects between developmental domains; a treatment-by-domain interaction across the nine analysable domains was not significant (χ²=8.72, 8 df; p=0.37) but has little power at this sample size, and the pattern therefore requires replication. After control of the FDR, no individual domain retained q<0.05 on either co-primary representation; Language 1 and Language 2 gave q=0.060 on the Subdomain quotient and q=0.155 on performance-age. The only outcome to meet the threshold was the language composite, on both the Subdomain quotient (q=0.006) and performance-age (q=0.026). Language 1 (VI) was the main individual contributor. The supportive IV-V-VIII cognition composite was directionally favourable but not statistically significant. All three analysable motor domains were null across representations, consistent with the parent-trial null motor DQ result.^15^ The pattern is biologically coherent: a nutritional intervention sufficient to shift mental DQ by about two points might be expected to manifest at 9–12 months, when communication functions are developing. Mechanistically, one-carbon metabolism responds to multiple B-vitamin and methyl-donor inputs and shapes fetal DNA methylation implicated in neurodevelopmental programming.^24^

A difference of 2.80 Subdomain quotient points corresponds to a standardised effect of 0.26 (95% CI 0.09 to 0.43), and to approximately 0.25 months, about a week, of language performance-age at a mean assessment age of 9.7 months. No minimal clinically important difference has been established for the Subdomain quotient, for the AEAR or for any derived DASII domain measure, because these are exploratory re-expressions of item-level content clusters rather than validated subscales with published norms.

Estimates at the two sites were directionally consistent and the interaction gave no evidence of heterogeneity, so we do not interpret the analysis as showing a site-specific effect.

### Strengths and limitations

Although DASII subdomain analyses were not prespecified outcomes in the original MATCOBIND protocol,^14^ the principles of this secondary analysis were agreed before group-level results were examined; the representations and the final composites were settled during the analysis, and the findings are exploratory. Four representations of domain performance (raw score, AEAR, performance-age and Subdomain quotient) were reported, but they are re-expressions of the same item responses, so inference rested on the two co-primary representations, with the raw score and AEAR as descriptive checks. Multiplicity was controlled with BH-FDR within the domain and composite families.^19^ DASII content domains were treated as exploratory profiling representations rather than new psychometric scales.^16^ Composites reduced multiplicity and aligned analyses with developmentally coherent functional families. The analysis also inherits key strengths of the MATCOBIND randomised controlled trial, including blinded developmental assessment with high inter-rater agreement.^15^

The parent trial was not powered for multiple domain-level comparisons, so these analyses remain hypothesis-generating. Several DASII domains required a priori exclusion because of ceiling or floor effects in the 9–12 months window. Raw score, performance-age, AEAR and Subdomain quotient are not psychometrically validated subscales. Site-stratified analyses were underpowered. The DASII was standardised in Indian infants and has not been separately validated in Nepal; the cultural proximity of the two populations and the examiner-administered, observational format of the instrument limit the scope for differential item functioning across sites, but we did not test for it and this qualifies a finding that concerns language specifically. Loss to follow-up was associated with poorer maternal baseline vitamin B_₁₂_ status, so the analytical cohort is modestly better nourished than the randomised cohort, although this could potentially attenuate rather than exaggerate a supplementation effect. Participant-level biomarker-domain modelling was not performed and is the next analytical step. Finally, assessment at 9–12 months captures only an early phase of communicative development, and the supportive memory and social-imitation signals did not reach statistical significance.

### Context within the existing evidence base

Our findings sit within a mixed evidence base on maternal B_₁₂_ supplementation and offspring neurodevelopment.^6–8^ The South Indian pregnancy-and-early-lactation trial of 50 µg/day reported no uniform neurodevelopmental benefit at 9 months but found higher expressive language scores at 30 months (β=0.14, p=0.03), alongside negative associations between elevated maternal homocysteine and expressive-language and gross-motor domains.^9,10^ Long-term follow-up at 72 months, including event-related potential measures, did not show a sustained neurodevelopmental benefit, suggesting that early language signals may be subtle and their trajectory uncertain.^25^ The PRIYA preconception trial (2 µg/day from preconception through pregnancy) showed better Bayley-III cognition and language DQ scores at 24–42 months in offspring of women who received vitamin B_₁₂_ alone, with no benefit when B_₁₂_ was combined with multiple micronutrients.^11^ In contrast, the Nepalese community-based placebo-controlled trial of 50 µg/day B_₁₂_ supplementation from early pregnancy until 6 months post partum did not improve Bayley-III cognitive composite scores at 6 or 12 months despite a substantial improvement in maternal B_₁₂_ biomarkers.^26^ A secondary analysis from that trial also reported no favourable effect on early motor performance and unexpectedly raised the possibility of a detrimental effect on motor scores, underscoring the importance of examining domain-level outcomes rather than relying only on composite scores.^27^ Differences in population (eg, from a community setting vs tertiary-care centre), comparator groups (eg, placebo control vs lower dose B_₁₂_ supplementation group), outcome measures (eg, Bayley-III cognitive composite DQ scores vs DASII mental/motor DQ scores and domains) and timing of cognitive assessments could all contribute to divergence between these trials.^6–8,28^ The 2024 Cochrane review highlighted the high heterogeneity between previous trials and judged current trial evidence on maternal B_₁₂_ supplementation and child cognitive or language outcomes to be of low to very low certainty.^8^ Observational cohorts from India, Singapore and Spain support associations between lower maternal vitamin B_₁₂_ status in pregnancy with poorer offspring cognitive and language outcomes,^12,13,29^ and newer cohort evidence links higher prenatal B_₁₂_ intake with better early language measures and verbal IQ.^30^ Against this background, the MATCOBIND trial contributed a pregnancy-initiated randomised trial in a predominantly vegetarian, nutritionally vulnerable South Asian population,^15^ and the present analysis suggests that its early cognitive phenotype was language-centred. This profile is consistent with longitudinal Indian observational data associating maternal B_₁₂_ during pregnancy with offspring cognition^29^ and with newer cohort evidence linking higher prenatal B_₁₂_ intake to better child speech development and verbal IQ at 5 and 8 years.^30^

### Implications and future directions

The language-centred pattern observed with the higher dose at 9–12 months now needs testing for persistence. Longer-term follow-up of the MATCOBIND participants and of other similar studies should include validated measures of receptive and expressive language. Participant-level mediation analyses should examine whether the language signal is explained by changes in maternal B_₁₂_ markers during pregnancy, and whether mediation varies by baseline deficiency. Replication trials may wish to prespecify language outcomes and measure concurrent maternal micronutrient status so that co-deficiency-mediated effect modification can be formally evaluated.

If replicated, these findings would suggest that developmental surveillance relying on composite scores alone may be insensitive to domain-specific nutritional effects. Bundled multi-micronutrient strategies warrant rigorous evaluation in settings where coexisting deficiencies are common. The optimal maternal dose for neurodevelopmental benefit remains an open policy question and should be addressed in trials incorporating a placebo or physiological-dose comparator.^26^

## Conclusions

In this exploratory secondary analysis of the MATCOBIND trial, between-arm differences in infant neurodevelopment at 9–12 months were concentrated in language-related domains, and the language composite was the only outcome to meet FDR control, suggesting a language-centred pattern that requires replication. The difference was approximately a quarter of a SD, its clinical importance needs further exploration and the measures on which it rests are derived rather than validated subscales. Future follow-up and replication trials of maternal B_₁₂_ supplementation should prioritise language outcomes to test persistence, clarify clinical relevance and inform maternal micronutrient supplementation policy.

## Supplementary material

10.1136/bmjph-2026-006254 online supplemental file 1

## Data Availability

Data are available upon reasonable request.
