# Supplementary material for: Higher-dose maternal vitamin B₁₂ supplementation and infant neurodevelopmental subdomains: a secondary analysis of the MATCOBIND randomised controlled trial from India and Nepal
Source: BMJ Public Health. 2026 Nov 9;4(4):e006254. doi: 10.1136/bmjph-2026-006254 (PMC13756379; doi:10.1136/bmjph-2026-006254)
Supplement: Supplementary file 1 [file bmjph-4-4-s001.docx]

**Supplementary material**

*Higher-dose maternal vitamin B₁₂ supplementation and infant neurodevelopmental subdomains: a secondary analysis of the MATCOBIND randomised controlled trial from India and Nepal*

Contents. Supplementary Tables S1 to S4.

**Supplementary Table S1. Ceiling and floor proportions for all fifteen DASII content domains in the analytic cohort (n=531)**

| **Domain** | **Mean (SD) raw score** | **Range** | **Infants at maximum, n (%)** | **Infants at minimum, n (%)** | **Distinct values** | **Status** |
| --- | --- | --- | --- | --- | --- | --- |
| Motor I (Neck control) | 6.99 (0.11) | 5–7 | 527 (99.2) | 1 (0.2) | 3 | Excluded |
| Motor II (Body control) | 19.09 (1.21) | 16–21 | 66 (12.4) | 13 (2.4) | 6 | Analysed |
| Motor III (Locomotion-1) | 4.09 (0.45) | 2–8 | 2 (0.4) | 1 (0.2) | 7 | Analysed |
| Motor IV (Locomotion-2) | 0.00 (0.00) | 0–0 | 531 (100.0) | 531 (100.0) | 1 | Excluded |
| Motor V (Manipulation) | 13.52 (0.83) | 9–14 | 366 (68.9) | 1 (0.2) | 6 | Analysed |
| Mental I (Visual cognisance) | 24.90 (0.32) | 23–25 | 479 (90.2) | 3 (0.6) | 3 | Excluded |
| Mental II (Auditory cognisance) | 7.00 (0.00) | 7–7 | 531 (100.0) | 531 (100.0) | 1 | Excluded |
| Mental III (Reaching and manipulation) | 27.29 (1.16) | 22–32 | 1 (0.2) | 1 (0.2) | 11 | Analysed |
| Mental IV (Memory) | 9.33 (0.74) | 6–10 | 247 (46.5) | 2 (0.4) | 5 | Analysed |
| Mental V (Social interaction and imitation) | 14.87 (1.73) | 11–21 | 1 (0.2) | 1 (0.2) | 11 | Analysed |
| Mental VI (Language 1) | 6.59 (0.75) | 4–9 | 3 (0.6) | 7 (1.3) | 6 | Analysed |
| Mental VII (Language 2) | 2.92 (1.21) | 0–7 | 1 (0.2) | 2 (0.4) | 8 | Analysed |
| Mental VIII (Understanding relationships) | 0.98 (0.28) | 0–4 | 2 (0.4) | 20 (3.8) | 4 | Analysed |
| Mental IX (Differentiation by use, shape and movement) | 1.78 (0.82) | 0–4 | 17 (3.2) | 11 (2.1) | 5 | Excluded |
| Mental X (Manual dexterity) | 0.01 (0.12) | 0–2 | 2 (0.4) | 529 (99.6) | 2 | Excluded |

Domains were excluded where more than 95% of infants were at the maximum or minimum attainable value, where fewer than five items were scorable in the 9 to 12 month window, or where the allocation-masked distribution showed negligible between-infant variability; Mental I (90.2% at the maximum, three distinct values) was excluded on this last criterion. Two retained domains, Manipulation (Motor V) and Memory (Mental IV), show appreciable ceiling effects (68.9% and 46.5% at maximum).

**Supplementary Table S2. Maternal characteristics at enrolment: women included in the analytic cohort versus women not included**

| **Characteristic** | **Included (n=531)** | **Not included (n=177)** | **p** |
| --- | --- | --- | --- |
| New Delhi site, n (%) | 283 (53.3) | 87 (49.2) | 0.385 |
| Head-of-household education, graduate or above, n (%) | 299 (56.3) | 97/176 (55.1) | 0.850 |
| Primigravida, n (%) | 318 (59.9) | 100/176 (56.8) | 0.529 |
| No meat or fish intake, n (%) | 394 (74.2) | 127 (71.8) | 0.588 |
| Age, years, mean (SD) | 28.11 (4.61) | 28.01 (4.77) | 0.809 |
| Gestational age at enrolment, weeks, mean (SD) [n=176] | 9.08 (1.95) | 8.78 (1.91) | 0.073 |
| Pre-pregnancy body mass index, kg/m², mean (SD) [n=176] | 23.29 (3.78) | 23.18 (3.49) | 0.731 |
| Height, cm, mean (SD) [n=176] | 156.79 (6.06) | 157.13 (6.03) | 0.514 |
| Serum vitamin B₁₂, pg/mL, mean (SD) [n=173] | 223.99 (117.40) | 198.76 (103.50) | 0.007 |
| Vitamin B₁₂ below 203 pg/mL, n/N (%) | 290 (54.6) | 113/173 (65.3) | 0.017 |
| Homocysteine, µmol/L, mean (SD) [n=173] | 11.36 (6.71) | 13.15 (7.92) | 0.008 |
| Holotranscobalamin, pmol/L, mean (SD) [n=173] | 39.18 (32.10) | 31.88 (27.69) | 0.004 |
| Haemoglobin, g/L, mean (SD) [n=173] | 122.05 (11.86) | 124.02 (12.97) | 0.078 |

p values from χ² tests with continuity correction for categorical variables and Welch t tests for continuous variables; hypothesis tests are appropriate here because inclusion was not randomised. Laboratory values are first-trimester serum concentrations and were available for 173 of the 177 women not included. Bracketed denominators apply to the not-included column: head-of-household education, primigravidity, gestational age at enrolment, pre-pregnancy body mass index and height were each missing for one woman not included.

**Supplementary Table S3. Sensitivity analyses for the language composite (250 µg/day minus 50 µg/day)**

| **Analysis** | **n** | **Subdomain Quotient, β (95% CI)** | **p** | **Performance-age, β (95% CI)** | **p** |
| --- | --- | --- | --- | --- | --- |
| Complete case (primary analysis) | 531 | 2.80 (0.95 to 4.65) | 0.003 | 0.25 (0.05 to 0.44) | 0.013 |
| Inverse probability of inclusion weighting, robust standard errors | 531 | 2.82 (0.99 to 4.66) | 0.003 | – | – |
| Multiple imputation by chained equations (m=200), all 708 randomised dyads | 708 | 2.80 (0.93 to 4.66) | 0.003 | – | – |

Inverse probability weights were derived from a logistic model of DASII follow-up on allocation, site, maternal age, gestational age at enrolment, baseline serum vitamin B₁₂, homocysteine, holotranscobalamin, haemoglobin, pre-pregnancy body mass index and primigravidity. Multiple imputation used the same predictors, imputing the language composite Subdomain Quotient for the 177 dyads without follow-up, with Rubin’s rules for combination. Attrition analyses were run on the Subdomain Quotient only.

**Supplementary Table S4. Unadjusted p values and Benjamini-Hochberg q values for every analysed domain and composite on the two co-primary representations**

| **Family (k)** | **Domain / composite** | **Subdomain Quotient: β (95% CI)** | **p** | **q** | **Performance-age: β (95% CI)** | **p** | **q** |
| --- | --- | --- | --- | --- | --- | --- | --- |
| Motor (k=3) | Body control (II) | 0.51 (−1.80 to 2.82) | 0.665 | 0.961 | 0.03 (−0.19 to 0.25) | 0.787 | 0.787 |
|  | Locomotion-I (III) | 0.03 (−1.29 to 1.36) | 0.961 | 0.961 | −0.03 (−0.15 to 0.10) | 0.682 | 0.787 |
|  | Manipulation (V) | 1.74 (−0.90 to 4.39) | 0.196 | 0.589 | 0.15 (−0.10 to 0.39) | 0.234 | 0.703 |
| Mental (k=6) | Reaching & manipulation (III) | 0.60 (−1.93 to 3.12) | 0.641 | 0.770 | 0.05 (−0.24 to 0.34) | 0.740 | 0.888 |
|  | Memory (IV) | 1.57 (−0.14 to 3.28) | 0.073 | 0.145 | 0.13 (−0.02 to 0.27) | 0.082 | 0.164 |
|  | Social interaction (V) | 1.05 (−0.63 to 2.72) | 0.220 | 0.329 | 0.07 (−0.10 to 0.23) | 0.420 | 0.630 |
|  | Language 1 (VI) | 3.06 (0.62 to 5.50) | 0.014 | 0.060 | 0.27 (0.02 to 0.51) | 0.031 | 0.155 |
|  | Language 2 (VII) | 2.55 (0.40 to 4.70) | 0.020 | 0.060 | 0.22 (−0.001 to 0.45) | 0.052 | 0.155 |
|  | Understanding relationships (VIII) | 0.30 (−2.25 to 2.84) | 0.820 | 0.820 | 0.02 (−0.23 to 0.27) | 0.895 | 0.895 |
| Composite (k=2) | Language (VI-VII; primary) | 2.80 (0.95 to 4.65) | 0.003 | 0.006 | 0.25 (0.05 to 0.44) | 0.013 | 0.026 |
|  | Cognition (IV-V-VIII; supportive) | 0.97 (−0.48 to 2.42) | 0.190 | 0.190 | 0.07 (−0.06 to 0.20) | 0.294 | 0.294 |

q values are computed within each family and separately for each representation by the Benjamini-Hochberg step-up procedure: for the ranked p values p(1) ≤ … ≤ p(k), q(i) = min over j ≥ i of p(j)·k/j. The decision rule for the primary outcome was conjunctive: the language composite was regarded as meeting the threshold only if q<0.05 on both representations. Raw score and AEAR are descriptive representations and were not entered into false discovery rate families.
